# Neuropsychological Sequelae of Carotid Angioplasty with Stent Placement: Correlation with Ischemic Lesions in Diffusion Weighted Imaging

**DOI:** 10.1371/journal.pone.0007001

**Published:** 2009-09-10

**Authors:** Laura Tiemann, Jutta Hubertina Reidt, Lorena Esposito, Dirk Sander, Wolfram Theiss, Holger Poppert

**Affiliations:** 1 Neurologische Klinik und Poliklinik der Technischen Universität München, Munich, Germany; 2 Medizinische Klinik der Technischen Universität München, Munich, Germany; University of Granada, Spain

## Abstract

**Background and Purpose:**

Few studies investigated the neuropsychological outcome after carotid angioplasty with stent placement (CAS), yielding partially inconsistent results. The present investigation evaluated the effect of CAS in patients with high-grade stenosis and assessed the predictive value of ischemic lesion number for postinterventional cognitive deterioration.

**Methods:**

22 patients were tested neuropsychologically before and six weeks after CAS. Cerebral ischemic changes were assessed with diffusion weighted imaging (DWI) prior to and after angioplasty.

**Results:**

Pre- to postinterventional cognitive performance improved significantly in terms of verbal memory (t = −2.30; p<0.05), whereas significant deterioration was noted regarding verbal memory span (t = 2.31; p<0.05). 8 (36%) persons conformed to the criteria of cognitive improvement. 6 patients (27%) were postinterventionally classified as having deficits. Analysis yielded no statistically significant correlations between lesion quantity and cognitive change.

**Conclusion:**

Both improvement and deterioration of cognitive functioning was observed in our collective of patients, leaving the neuropsychological outcome after percutaneous transluminal angioplasty unpredictable in individual cases. The presence of acute ischemic lesions on DWI was found to be not tightly associated with cognitive dysfunction after CAS.

## Introduction

High-grade carotid stenosis as well as asymptomatic narrowing of the carotid arteries due to atherosclerosis may cause global cerebral hypoperfusion and thus result in cognitive dysfunction [1]–[4]. A regularization of the cerebral perfusion however is thought to be associated with improved cognitive functioning [5], [6]. On the other hand, surgical interventions like carotid endarterectomy (CEA) as well as endovascular treatments like percutaneous transluminal angioplasty (PTA) or carotid angioplasty with stenting (CAS) hold a risk of subsequent complications and might lead to cognitive deterioration as well. The emergence of PTA in 1974 initiated a debate concerning alternative procedures to carotid surgery. Although the applied materials and techniques have been dramatically improved since that time [7], the optimized choice in methods is currently still being evaluated [8]–[11]. Whereas the advantages of endovascular interventions over CEA include shorter hospitalization and an avoidance of minor surgical complications, which occur in about 10% in case of CEA [12], carotid PTA has been criticised for causing significantly more microembolism as inferred from transcranial Doppler [13], [14]. In the context of cardiac surgery, there is some evidence that neuropsychological outcome is related to microembolic load [15]. Accordingly, one might suggest that carotid PTA might cause disproportionate cognitive impairment in comparison to CEA. A recently conducted study by Witt and colleagues [16] assessed the neuropsychological outcome after CEA and CAS in 24 and 21 patients, respectively, who were assigned randomly to the different treatments. Both groups achieved comparable results on the neuropsychological testbattery that was administered before, as well as 6 and 30 days after intervention. Accordingly, CAS was not found to be associated with greater cognitive deterioration than CEA. These results agree well with the empirical data conveyed by an other study, reporting no significant group differences in the cognitive domain when assessed six weeks and six months after CAS and CEA, respectively [17].

Studies evaluating the precise extent to which cognitive functioning is altered after PTA and CAS are rare, and report at least partially inconsistent findings. Lehrner and colleagues [18] compared 20 patients suffering from high-grade carotid stenosis before and 6 months after unilateral stent-protected carotid angioplasty with regard to their neurocognitive status. Using reliable change indices methodology to evaluate the extent of change after the intervention, the authors concluded that angioplasty with stenting did not change the cognitive functioning significantly when referring to the whole group. For some patients, however, significant improvement or deterioration in domains of concentration, sustained attention, psychomotor speed, verbal fluency and cognitive interference could be noted. Xu and colleagues [19] compared the cognitive status of 54 patients undergoing CAS to a matched control group with similar medical condition requiring carotid angiography. One week as well as 12 weeks after intervention, the patients that underwent CAS showed significantly more pronounced improvement on measures of verbal memory. The authors suggest correction of cerebral hypoperfusion and reduction of artery-to-artery embolization responsible for this improvement after stenting. In a recently conducted study by Turk et al. [20] 17 patients assigned to carotid stent placement were assessed in terms of their cognitive function using the Mini-Mental State Examination, an extended mental status examination, a subjective cognitive status measure, and a psychomotor performance test for speed. Differences in test scores were calculated before and 3 months after stent placement and tested for significance. Whereas no significant change was noted in scores from Mini-Mental State Examination or in the speed of psychomotor performance, the scores from the extended mental status examination did improve significantly.

Mlekusch and colleagues [21] investigated the effect of protected carotid artery stenting on neurocognitive function in 71 patients with particular consideration of the angiographic filling of the ipsilateral anterior cerebral artery. To this end, neuropsychological tests of frontal lobe function were administered prior to and 6 months after carotid artery stenting. In agreement with the findings of Lehrner et al. [18] significant improvement of neuropsychological performance was evident in a circumscribed proportion of patients (45%). Angiographic filling of the anterior cerebral artery could be identified as a factor that significantly differentiated between patients who profited by the intervention in manners of cognitive function and those who did not, suggesting the amelioration of frontal lobe perfusion to account for the improvement in cognition.

Despite the augmented frontal perfusion is thought to account for some variance in cognitive outcome after endovascular intervention, the proper neurophysiological substrate determining cognitive improvement, deterioration or stability remains unclear. Especially in consideration of the finding that alterations of cognitive status apply to a certain assembly of persons merely [18], [21], it needs to be clarified which peri -or postinterventional conditions are reliably associated with cognitive alterations to one or the other direction. An approach in this context aims to correlate the number and size of postinterventional ischemic lesions with the clinical outcome. Combined with the identification of risk factors in developing acute ischemia during intervention this approach promises a more adequate assignment of patients to treatments and hence a minimization of consequential (cognitive) damage [22].

In case of imaging acute stroke after endovascular treatment, diffusion weighted imaging (DWI) turned out to be ideally suited. This is due to its ability to quantitatively demonstrate cerebral ischemia within minutes after its onset [23] and to differentiate between ischemic versus non-ischemic and acute versus chronic lesions, respectively [24]. Contrasted with conventional magnetic resonance imaging, DWI further entails less false negative results [25]. Although parts of the lesions displayed by DWI techniques are clinically mute in terms as they lack a clinical correlate [26], there is evidence suggesting lesion volumes determined by DWI to be predictive of clinical severity and outcome [25], [27], [28]. Few investigations agree to the insistence of an equivalent correlation of acute ischemic lesions and neuropsychological outcome parameters [29]. However, evidence is rare and a potential coherence of lesion volume and cognitive outcome needs to be assessed.

As an interim summary, it may be pointed out that some investigations are currently pleading for potential cognitive deterioration after endovascular treatment of carotid stenoses [16], [17]. Some studies, however, provide evidence for an improvement of neuropsychological status in at least certain cognitive domains [19], [20] or in a specific collective of patients [18], [21]. Further, factors that are able to distinguish between persons whose cognitive ability will benefit or remain unaffected from the treatment and those whose neuropsychological status might worsen remain widely unknown. Accordingly, further research is needed to define the specific value of endovascular interventions in terms of neuropsychological outcome. The present investigation was conducted with the objective to further evaluate the effect of PTA in sample of patients with high-grade stenosis. Moreover, as it can not be stated with certainty if cognitive deterioration after endovascular treatment correlates with the number and size of ischemic lesions detected by DWI, the present study is the first to investigate the potential neurophysiological substrate of postinterventional cognitive damage by associating cognitive status with ischemic lesion quantitiy.

## Methods

### Patients

22 patients with asymptomatic stenosis of the left (n = 10) or right (n = 12) internal carotid artery undergoing PTA were recruited at facilities of the Technische Universität München, namely the *German Heart Center* and the *Interdiciplinary Vascular Center* of the university hospital rechts der Isar in Munich. Routine MRI exclusion criteria were applied. All patients completed the follow-up as scheduled. The severity of carotid stenosis was evaluated by measuring peak systolic velocity (PSV) with angle correction at the narrowest point of the stenosis. The degree of stenosis was classified as mild (200 cm/sec), moderate (200–299 cm/sec) or severe (>300 cm/sec, or a decrease of PSV combined with distinct duplex sonographic signs of filiform stenosis). All patients showed a relevant increase in blood flow velocity (PSV greater than 200 cm/sec). The collective consisted of 2 female and 20 male patients. The mean age of the participants was 67.8 (SD = 8.18) years, with the youngest patient being 55 and the oldest patient being 83 years of age. Premorbid intelligence averaged 107 (SD = 10.5) when estimated using a German vocabulary test (“Mehrfach-Wahl-Wortschatz-Test”, MWT-B; [30]). All patients had normal or corrected-to-normal visual acuity.

The protocol was approved by the local ethical committee at the Technische Universität München. All patients gave their informed consent prior to participation.

### Procedure

Patients were appointed to the *German Heart Center Munich* one day prior to the scheduled intervention and stayed hospitalized for three to four days provided that the intervention proceeded without complications.

Patients underwent DWI three to one day prior, as well as one day after angioplasty.

Baseline neuropsychological testing was administered three to one days prior to the treatment. Neuropsychological deficits become relevant for the affected patients only if they are not transient, but long lasting and therefore evident in every day life. Accordingly, a correlation between the rather short-lived DWI lesions and lasting cognitive deterioration would indicate a predictive value of diffusion weighted imaging for the cognitive status accordingly, being of practical importance for therapeutic and diagnostic decision making. Thus, the follow-up evaluation on cognitive function took place after at least 6 weeks from PTA.

### Neuropsychological assessment

Neuropsychological testing at baseline and follow-up was accomplished by an experienced neuropsychologist at the department of psychiatry of the university hospital rechts der Isar, Munich. Cognitive functions from the domains of (working) memory, attention and mental speed, executive function and motor coordination were included in testing. A German vocabulary test [30] was used to estimate premorbid general intelligence level and was administered prior to the procedure only. The MWT-B consists of 37 sets of five words, each set comprising one genuine word and four nonsense words. The subject's task is to identify (but not to define) the genuine word in each set. An IQ score is calculated from the number of correct responses in relation to the subject's age.

A list learning test [31] was used to assess short- and long-term memory function. In this test, a list consisting of 8 word-stimuli is verbally presented to the subjects. Immediately after the presentation subjects are advised to reproduce as many words as possible. 20–30 minutes later, the patients are further asked to recognize the 8 test stimuli out of 8 distractor-stimuli. The number of reproduced as well as correctly recognized words is translated in an age-specific score for analysis.

A number-connecting-test [32] was administered to evaluate information processing speed. The test requires the patients to connect numbers ranging from 1 to 90 in numerical order as fast as possible. The time needed to complete four test sheets successively is averaged and considered for analysis.

Verbal memory span and working memory function was measured by the subtest *digit span forward* and *backward* from the revised German version of the Wechsler Adult Intelligence Scale [33]. This test requires the repetition of an increasing number of digits in both correct and converse sequence. Visual memory span and working memory was assessed by the subtest *spatial span forward* and *backward* from the revised German version of the Wechsler Adult Intelligence Scale [33]. Analogous to testing in verbal modality, the visual subtest requires the repetition of a demonstrated block-tapping sequence with increasing length in forward and backward condition. The correct answers are accumulated, z-transformed and then serve as parameter for the analysis of verbal/visual memory span and verbal/visual working memory, respectively.

In terms of assessing executive functioning, a verbal fluency task was accomplished [34]. Phonemic (*p-words*) and semantic (*animals*) fluency tests were administered. The time limit for generating words is restricted to one minute in both the phonemic and semantic condition. A z-score may be calculated from the number of correctly generated words allowing for the subject's age.

The results of a block-design-test from the revised German version of the Wechsler Adult Intelligence Scale [33] served as parameter for visuo-motorical coordination. Subjects are instructed to reproduce seven two-dimensional patterns by manipulating 16 wooden cubes. In defining the z-score for the statistical analysis, the accuracy as well as the time needed to complete the tasks is considered.

### Percutanous transluminal angioplasty

Angiography of the aortic isthmus was done in all patients for diagnostic purposes prior to intervention. PTA with stenting was performed in the same session under local anesthesia via percutaneous transfemoral access with a long sheath. After placing the sheath in the Arteria carotis interna, the stenosis was predilated by using a balloon catheter ranging from 3.0 to 4.0 millimeters in diameter. Monorail systems were used for both balloon dilatation and stent implantation. The final outcome of angioplasty and stenting was documented angiographically. All PTA were performed by an experienced angiologist and an experienced interventional radiologist. No protection devices were used. The stenosis was predilated before stent placement.

One day prior to intervention, patients were supplied with 600 mg acetylsalicylic acid (ASS) in purpose of anticoagulation. On the day of intervention, a loading dose of 900 mg Clopidogrel as well as 130 IU Heparin/kg body weight was administered additionally. Patients highly susceptible for anxiety were supplied with a sedative. During intervention 3000 IU of heparin were applicated intraarterially to increase the activated clotting time. In order to prevent bradycardia, the predilatation occurred after intravenuous application of atropine in a dosage of 0.5–1.0 mg. After intervention, ASS and Clopidogrel were administered for the duration of 4 weeks in a dosage of 200 mg and 75 mg, respectively. Following 4 weeks, either of these substances was chosen for long-term-medication.

### Diffusion weighted imaging

MRI-studies were performed on a 1.5 T whole body imaging system with a head coil (Magnetom Symphony Quantum gradient, Siemens Medical Systems, Germany). Whole brain DWI was carried out with an isotropic echo planar sequence. Sagittal, coronal and transversal studies were obtained, each with b values of 0, 500 and 1000 s/mm^2^, TR 4006 ms, TE 83 ms, quantum gradient 30 mT/m, slew rate 125 mT/m/ms, rising time 240 microseconds, slice thickness 4–6 mm, gap 1.5 mm, 128×128 matrix and 220×220 mm field of view. The diffusion-weighted data was automatically processed by the scanner's software (Numaris^©^ 3.5) in order to minimize the effects of diffusion anisotropy.

ADC maps were also automatically processed by the scanner's software.

The MRI was conducted with special consideration of the number and location of any lesions. All images were re-analyzed by two experienced neuroradiologists who were blinded to clinical details including the kind of intervention. An acute ischemic lesion in DWI was only diagnosed if increased signal intensity was visible at least on two planes and if a corresponding decreased signal intensity was detected in the apparent diffusion coefficient (ADC) image. The diagnosis of the lesion was only confirmed if the lesion was not seen in the preprocedural films and if both neuroradiologists agreed on the DWI findings. Manually tracing the lesions with the internal measuring function of the magnetic resonance imaging scanner and multiplying the area by slice thickness obtained volume size. These volumes were added up plane by plane to get the entire volume of one lesion. However, many lesions were smaller than the selected slice thickness. In this case one of the other planes was also used to determine the precise lesion dimension.

### Statistical analysis

Statistical analyses were performed using SPSS for windows (release 16.0.1, SPSS Inc., Chicago).

In the context of descriptive data analysis, means and standard deviations were calculated for the whole group considering each neuropsychological test result in baseline and post-treatment condition.

As a first step in inductive statistics, the normal distribution for all test results was verified using the Kolmogorov-Smirnov test. In case of normal distribution, the extent of pre- to post-treatment difference in cognitive status was assessed by applying t-tests for dependent samples. The Wilcoxon signed-ranks test for two dependent samples was used if the named premises were violated.

To answer the question as to how many patients cognitively declined or improved, we adopted the conventional definition of substantial neuropsychological alteration that has been widely used in studying the impact of cardiac surgery [17], [18], [21]. The standard deviation for each test was computed from preoperative test scores. A cognitive change was assumed if the postprocedural test results differed from the preinterventional performance in two or more domains, and if these differences exceeded one standard deviation each.

To assess the impact of postinterventional ischemic lesions on cognitive outcome, difference values of pre- and postinterventional performance were calculated for all subjects and neuropsychological tests (score_preprocedural_ – score_postprocedural_). Improved performance in any test is revealed by a lower score. Because of the potential for all neuropsychological tests to show learning with repetition, a greater improvement in neuropsychological scores in this analysis will reflect a combination of greater learning and less deficit [35], [36]. The collective was then divided in two groups (group 0: No postinterventional lesions detected with DWI, group 1: Postinterventional lesions could be detected with DWI), to compare group-dependent difference values using t-tests for independent samples.

Further the correlation between pre- to postinterventional cognitive change and number of lesions in DWI was evaluated using Spearman's rank correlation test.

## Results

### Diffusion weighted imaging

Postoperative DWI revealed ipsilateral ischemic lesions in 10 (45%) out of 22 patients. The number of lesions varied across subjects from 1 to 9 lesions (M = 3.4; SD = 2.6). In all patients, lesions were detected ipsilaterally to the side of carotid stenosis. 4 patients showed contralateral lesions additionally.

### Neuropsychological status prior to intervention

**Figure 1:**
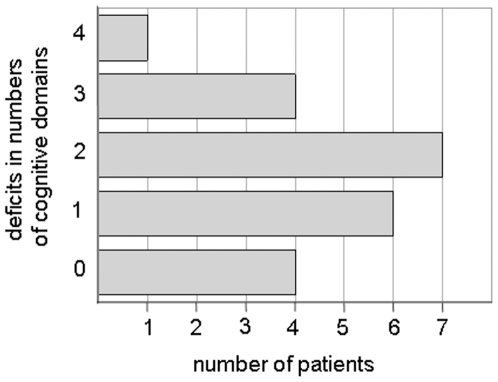
Displayed are the numbers of patients who showed cognitive deficits prior to intervention in 1, 2, 3 or 4 cognitive domains (z-score<−1.0).

To assess whether the patients showed any cognitive deficits prior to the intervention, we analyzed the pre-treatment cognitive data. A deficit in a cognitive domain was assumed when the patient's test score differed from the normative test data in one ore more standard deviation (z ≤−1). Group means of pre-treatment z-scores showed no cognitive deficit in any cognitive domain (all means of z-scores >−0.36). However, on single subject level, cognitive deficits could be noted for 18 patients in one or more cognitive domains. Information as to how many patients were neuropsychologically impaired prior to intervention in one, two, three or four domains can be extracted from figure 1.

### Neuropsychological changes after PTA (whole group)

Means, z-scores, standard deviations and results of applied t-tests for all neuropsychological tests in baseline and postprocedural assessment are displayed in table 1.

**Table 1:** Neuropsychological data as assessed prior and six weeks after PTA.

cognitive function		baseline		6 weeks		test statistic
		M (z-score)	SD	M (z-score)	SD	*t*
verbal memory		9.73 (−0.15)	2.55	10.81 (0.36)	2.36	−2.30*
memory span	*verbal*	8.45 (0.8)	1.6	7.59 (0.3)	1.79	2.31*
	*nonverbal*	7.32 (−0.36)	1.89	7.91 (0.02)	2.69	−0.97
working memory	*verbal*	6.77 (0.34)	2.45	6.55 (0.21)	2.26	0.54
	*nonverbal*	6.95 (−0.19)	1.27	7.45 (0.12)	1.99	−1.42
mental speed		31.52 (−0.06)	10.57	30.31 (0.00)	10.32	0.86
visuo-motor coordination		23.3 (1.15)	5.19	23.55 (1.21)	4.78	−3.12
phonemic verbal fluency	*p-words*	12.27 (−0.34)	5.94	14.95 (0.86)	4.72	−2.0^t^
semantic verbal fluency	*animals*	28.14 (−0.02)	5.45	28.45 (0.02)	3.95	−0.41

* p<0.05.

t p<0.10.

Applied t-tests met criteria for statistical significance in case of verbal memory (t = −2.30; p = 0.03) and verbal memory span (t = 2.31; p = 0.03). A trend though not statistically significant was found in case of phonemic verbal fluency (*p-words*; t = −2.0; p = 0.06).

**Figure 2:**
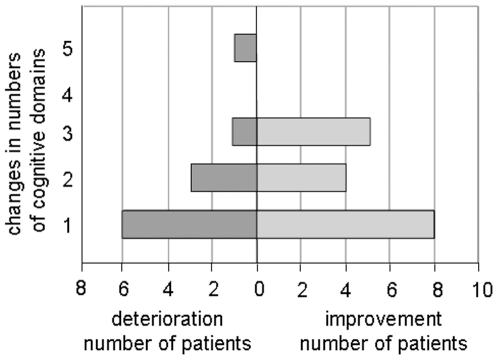
Displayed are the numbers of patients who cognitively deteriorated or improved 6 weeks after intervention in 1, 2, 3, 4 or 5 cognitive domains. Adopting a conventional definition, cognitive change is assumed as significant if it exceeds one standard deviation in two or more domains.

Analysis as to how many patients changed in cognitive status revealed that 9 persons met the described criteria for cognitive improvement, whereas 5 persons conformed to the criteria of cognitive deterioration (see figure 2). As there are 2 persons who improved in 2 or 3 cognitive domains, respectively, but changed for the worse in 3 and accordingly 2 domains concomitantly, one can assume 8 persons conforming to the criteria of cognitive improvement, and 6 persons satisfying the criteria of cognitive deterioration on a net basis.

### Neuropsychological changes after PTA (patients with DWI-lesions)

**Figure 3:**
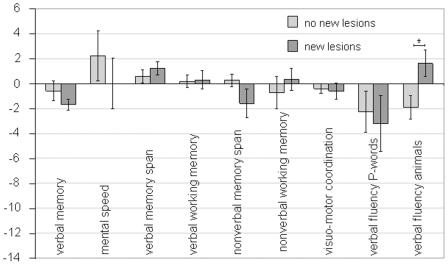
Comparison of the two groups with (dark grey) and without (light grey) new lesions in DWI in terms of the extent of pre-to postinterventional cognitive change. Statistical significances are indicated. * p<0.05.

Signal hyperintensities detected with diffusion weighted imaging were observed in 10 patients. Considering this subgroup selectively a statistically significant improvement in verbal short term memory after PTA became evident (t = −3.79; p = 0.004). Further a deterioration in verbal memory span was noted, which turned out to be a trend, though not statistically significant (t = 2.25; p = 0.051). A direct comparison of the pre- to postinterventional change score enables the effective evaluation of the extent to which both groups (with and without new lesions in DWI) changed in cognitive status (figure 3). The two groups differed significantly in terms of pre- to postinterventional cognitive change only in case of semantic verbal fluency (t = −2.49; p = 0.02).

### Correlation of cognitive change with diffusion weighted imaging

In the framework of our a priori hypothesis, we expected a higher quantity of new DWI lesions to be associated with greater postinterventional cognitive deterioration. The absence of new lesions detected with DWI was further assumed to be of predictive value for a stable cognitive performance or cognitive improvement. Regarding the group of patients with new DWI lesions selectively (n = 10), descriptive analysis revealed that only two of them conformed to the criteria of postinterventional decline. Four persons with newly acquired lesions remained stable regarding their neuropsychological test performance, whereas another two persons actually improved.

**Figure 4:**
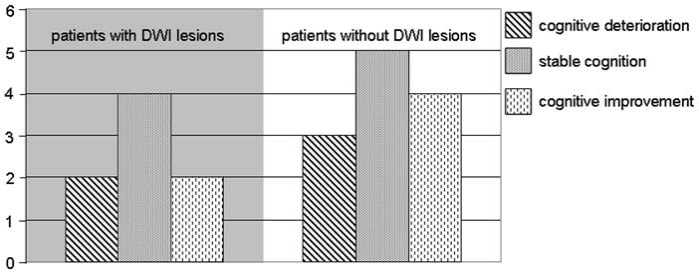
Number of patients who cognitively deteriorated, remained stable or improved after the intervention, separately displayed according to the incidence of new DWI lesions.

12 test persons did not exhibit any postinterventional hyperintensities as measured with DWI. In this group of patients, 9 persons remained stable or improved in matters of neuropsychological performance (n = 5 or n = 4, respectively). However, in case of 3 subjects a significant cognitive decline was denoted. Figure 4 summarises the results of descriptive analysis.

Spearmans rank correlation test was used to determine if a higher quantity of new lesions as detected with DWI was associated with a greater decline in cognitive status. Correlation coefficient reached level of significance only in case of phonemic verbal fluency (r = 0.47; p = 0.03), indicating higher numbers of new lesions after intervention to be associated with a better postinterventional performance, which is incompatible with our a priori hypothesis. Analysis yielded no further statistically significant correlations between lesion quantity and cognitive change. An overview of all correlation coefficients and test statistics is displayed in table 2.

**Table 2:** Displayed are means and standard deviations of the difference values for all cognitive domains, correlation coefficients and the probability values.

cognitive function		cognitive change score		correlation coefficient	probability value
		M	SD	r	p
verbal memory		−1.09	2.22	−.32	.15
memory span	*verbal*	.86	1.75	.18	.43
	*nonverbal*	−.59	2.87	−.31	.16
working memory	*verbal*	.23	1.97	.77	.77
	*nonverbal*	−.50	1.65	−.08	.71
mental speed		1.20	6.6	−.15	.51
visuo-motor coordination		−.25	3.58	−.08	.73
phonemic verbal fluency	*p-words*	−2.68	6.28	−.03	.88
semantic verbal fluency	*animals*	−.32	3.68	.47	.03

With exception of a significant association between semantic verbal fluency and lesion quantity, correlation analysis yielded no significant results.

## Discussion

Due to the advancements of the past years, percutaneous transluminal angioplasty with stenting of the internal carotid artery emerged as an alternative procedure to common carotid surgery. Although the advantages of PTA over CEA include shorter hospital stays, use of local anesthetics and lower risk of surgical trauma, the medical outcomes are comparable [37]. Thus, any significant differences in long-term cognitive functioning could influence therapeutic decision making.

The principal aim of this study was to evaluate the effect of PTA on pre- to postinterventional cognitive status. Further we put into question if newly accomplished lesions detected with DWI account for postinterventional cognitive deterioration.

Therefore, the performance of 22 patients in a neuropsychological test battery was evaluated three to one days prior to, and at least six weeks after planned PTA. Structural changes were assessed with DWI three to one days prior to intervention as well as one day after angioplasty.

The results comparing the pre- and postinterventional test performance reveal a significant change of status in two of nine cognitive domains: Regarding all 22 patients, an improvement in terms of verbal memory could be noted, whereas the performance in terms of verbal memory span had declined significantly after intervention. Further patients were able to retrieve more semantically related words from semantic memory after angioplasty, which turned out to be a statistical trend.

Cognitive change being defined as an alteration of one or more standard deviation in two or more cognitive domains, the analysis as to how many patients changed with regard to their cognitive function classified 8 patients as having significantly profited from intervention. Another 5 persons conformed to the criteria of cognitive deterioration. Interestingly, 2 persons fulfilled both the criteria for cognitive decline and cognitive improvement concomitantly.

These results suit well with the current scientific findings that report partly inconsistent or even conflicting effects of PTA on cognitive outcome [1]. For one, both cognitive improvement [19]–[21] as well as deterioration [17] are being reported in the literature and by this study. For another, the studies do not completely agree in terms of which cognitive domains are predominantly affected by the intervention. At least in parts, this might be attributed to the use of different or even insensitive neuropsychological measures. On the other hand, the variable deficit patterns found in different studies might be due to the absence of a typical *core deficit* that might be characteristic for carotid angioplasty. Consequently, one can assume that a change of cognitive status can not be expected after angioplasty in general, neither in terms of significant improvement, nor in terms of cognitive decline. However, alterations in neuropsychological test performance have become evident for a circumscribed collective of patients nearly consistently. Due to this, further research is needed to define factors that are able to distinguish between persons whose cognitive ability will benefit or remain unaffected from the treatment and those who are at a high risk to change to the worse. As long as such risk factors remain unidentified, the neuropsychological outcome continues to be unpredictable in individual cases.

Ischemic lesions emerging perioperatively might be able to discriminate between positive and negative cognitive outcome. A continuative analysis should therefore indicate if decline in cognitive functioning was restricted to those patients who showed acute ischemic lesions after angioplasty as assessed with DWI. New lesions were detected in 10 (45%) out of 22 patients. Similar to the analyses concerning the undivided group of 22 patients, comparisons of pre- and postinterventional cognitive status yielded a significant improvement of verbal memory performance in the group of patients with DWI lesions. Moreover, a trend towards degraded functioning was noted in case of verbal memory span. Comparing the extent to which both groups (with and without new DWI lesions) changed, they differed significantly only in one of the assessed cognitive domains. In addition, this difference in the extent of group-dependent cognitive change was incompatible with our a priori hypothesis: Whereas the patients with lesions improved in terms of semantic verbal fluency, deterioration was noted in the same domain for the group with no DWI lesions. The extent of cognitive change did not correlate with the number of lesions detected with DWI, except for one negative correlation between quantity of lesions and semantic verbal fluency, which was inconsistent with the a priori hypothesis. On an individual level, 80% of the patients in the lesions-group improved or remained unaltered with regard to their cognition despite of ischemia occurring during intervention. This leaves 20% of the patients in this group satisfying the criteria of postinterventional cognitive deterioration. Interestingly, in the non-lesion-group, cognitive deficits were identified in an even greater portion of patients (25%), raising the question of the neurophysiological substrate of the evident deficits, and how to sensitively detect them with available imaging methods.

An explanation for the lack of correlation between quantity of lesions and cognitive deficit might be the reversibility of DWI lesions after angioplasty that was recently demonstrated in a study by Hauth and coworkers [38]. About 97% of the lesions visible in DWI showed no manifestations at 6 month follow up and were clinically silent. Thus, these lesions do possibly correspond to a transient vascular etiology and are potentially of no sustained neurological or no neuropsychological sequelae. The hypothesis of clinically silent lesions is compatible with the findings of other investigators [26], [39], [40].

Recently, Wolf and colleagues [41] conducted a study to assess the predictive value of DWI lesions for brain infarction after CEA. The used devices and the settings during intervention were the same as in the study we presented here. Postoperative DWI revealed ipsilateral ischemic lesions in 15 patients. A T1 weighted MR scan performed 7–10 days after intervention revealed brain infarction in seven of these patients. However, postoperative neurological deficits were evident only in 2 patients. According to these findings, DWI can be assessed as sensitive method of demonstrating ischemic events after CEA. The strategic localization of ischemic lesions in non-eloquent brain areas, however, might explain the occurrence of no or only a transient deficit.

Moreover it can be assumed that the partially small lesions yielded a clinically very isolated and selective neuropsychological deficit. The test battery used in this study included predominantly measures of global cognitive functioning, such as verbal memory, psychomotor speed or verbal fluency. It can not be ruled out that some lesions classified as clinically silent actually caused a circumscribed cognitive deficit for which the tests used were too insensitive to detect.

As the amount of solid cerebral microemboli during carotid stenting does not relate to the frequency of silent ischemic lesions [42], [43] it is intriguing to assume the cognitive outcome to be more closely related to microembolism detected by transcranial Doppler than to new ischemic lesions visible in DWI. Although most researchers argue in favour of this hypothesis [38], [44], [45] there is evidence that there may be even other causes such as hemodynamic or metabolic stressors occurring after angioplasty accounting for postinterventional cognitive decline [16], [46]. A recent study by Heyer and colleagues [46] found neuropsychological dysfunction in the absence of structural evidence for cerebral ischemia after uncomplicated CEA. The result is in line with the findings of this study, having classified 25% of the patients without visible DWI lesions as postinterventionally cognitively deteriorated. The authors conclude that cerebral hypoperfusion, which is missed by DWI in the majority of cases, plays a more significant role in cognitive injury than microembolization.

Major limitations of the present study are the rather small sample size as well as the lack of a control group. Moreover, since no parallel versions of the psychometric tests were used, it can not be excluded that repetition of the tests had an influence on task performance. Further studies should control for these effects.

In summary, the neuropsychological outcome after percutaneous transluminal angioplasty continues to be unpredictable in individual cases. Both improvement and deterioration of cognitive functioning are observed in specific collectives of patients, whereas the neurophysiological substrate remains widely unspecified. The presence of acute ischemic lesions on DWI is not tightly associated with cognitive dysfunction after PTA. Further research needs to address the issue, whether lesion size and location, the insensitivity of the NP battery or both are responsible for this low correlation.
